# Research on High-Precision PGC Demodulation Method for Fabry-Perot Sensors Based on Shifted Sampling Pre-Calibration

**DOI:** 10.3390/s25195990

**Published:** 2025-09-28

**Authors:** Qun Li, Jian Shao, Peng Wu, Jiabi Liang, Yuncai Lu, Meng Zhang, Zongjia Qiu

**Affiliations:** 1 Electric Power Research Institute of State Grid Jiangsu Electric Power Co., Ltd., Nanjing 211102, China; qun_li@sina.com (Q.L.); 18851790705@163.com (J.S.); 15105168844@163.com (P.W.); ngsh@163.com (J.L.); 15105168845@163.com (Y.L.); 2 Institute of Electrical Engineering, Chinese Academy of Sciences, Beijing 100190, China; mengzhang@mail.iee.ac.cn

**Keywords:** phase-generated carrier, Fabry–Perot sensor, phase delay compensation, shifted sampling, nonlinear distortion, demodulation stability

## Abstract

To address the issues of quadrature component attenuation and signal-to-noise ratio (SNR) degradation caused by carrier phase delay in Phase-Generated Carrier (PGC) demodulation, this paper proposes a phase delay compensation method based on sampling-point shift pre-calibration. By establishing a discrete phase offset model, we derive the mathematical relationship between sampling point shift and carrier cycle duration, and introduce a compensation mechanism that adjusts the starting point of the sampling sequence to achieve carrier phase pre-alignment. Theoretical analysis demonstrates that this method restricts the residual phase error to within Δ*θ*_max_ = π*f*_0_/*f*_s_, thereby fundamentally avoiding the denominator-zero problem inherent in traditional compensation algorithms when *θ* approaches 45°. Experimental validation using an Extrinsic Fabry–Perot Interferometric (EFPI) ultrasonic sensor shows that, at a sampling rate of 10 MS/s, the proposed pre-alignment algorithm improves the minimum demodulation SNR by 35 dB and reduces phase fluctuation error to 2% of that of conventional methods. Notably, in 1100 consecutive measurements, the proposed method eliminates demodulation failures at critical phase points (e.g., π/4, π/2), which are commonly problematic in traditional techniques. By performing phase pre-compensation at the signal acquisition level, this method significantly enhances the long-term measurement stability of interferometric fiber-optic sensors in complex environments while maintaining the existing PGC demodulation architecture.

## 1. Introduction

Fiber optic interferometric sensors have emerged as a crucial sensing technology in extreme environments due to their high sensitivity, immunity to electromagnetic interference, wide bandwidth response, and fully passive optical characteristics. These sensors are widely used in acoustic precision measurement [1,2], underwater acoustic detection [3,4], vibration measurement [5,6,7,8,9], and online monitoring of partial discharge inside power equipment [10,11]. The technology takes measurements by converting external physical quantities into interferometric phase changes, where the performance of demodulation algorithms directly determines the system’s practicality and reliability. The Phase Generated Carrier (PGC) demodulation technique effectively suppresses low-frequency noise by modulating low-frequency sensing signals to high-frequency sidebands. Through mixing with constructed first and second harmonic carrier signals of the interference signal, followed by low-pass filtering and quadrature operations, it enables the recovery of interferometric phase information [12,13].

However, during signal transmission, acquisition, and digital processing, time delays are inevitably introduced in signal generation and processing, causing asynchronization between the carrier signal and received interference signal. This leads to problems such as demodulated quadrature component attenuation, signal amplitude reduction, loss of orthogonality, and noise gain amplification. Particularly when the delay reaches certain specific values, it may cause signal amplitude to approach zero and signal-to-noise ratio to collapse, becoming a key bottleneck restricting system performance [14].

To address this issue, researchers have proposed various compensation methods. Zhang [15], Volkov [16], and Xie [17] proposed a series of correction algorithms based on real-time delay extraction, which extract delay information from mixed signals and dynamically compensate it into the arctangent calculation. However, when approaching certain values, these methods cause the compensation coefficient calculation error to amplify dramatically as the denominator approaches zero, affecting demodulation accuracy. Huang [14], Li [18], Qu [19], and Qian [20] introduced phase compensation terms for optical fiber propagation delay in algorithms, achieving signal alignment by adjusting carrier phase to reduce errors. These methods rely on precise measurement of carrier phase delay and require stable working conditions; otherwise, the compensation effect is susceptible to environmental changes. Moreover, due to possible non-ideal effects in the system, such as accompanying amplitude modulation and device nonlinearity, even after compensation, there may still be residual nonlinear distortion affecting demodulation accuracy. Nikitenko [21] used quadrature operations to eliminate the influence of carrier phase delay; Peng [22], Ni [23], and Yan [24] proposed ellipse fitting-based methods that correct by analyzing the distribution pattern of demodulated signals in the sine-cosine plane. These methods have improved demodulation accuracy to some extent but typically involve complex mathematical operations or iterative algorithms, which may introduce additional computational burden in high-speed real-time measurement applications. Ma [25] proposed an improved PGC algorithm insensitive to carrier delay and modulation depth, enhancing system robustness though at some cost to sensitivity or linearity. Dong [26] and Yan [27] performed nonlinear error compensation through joint calculation of carrier delay and modulation depth, but due to system coupling factors, residual nonlinear distortion remains difficult to eliminate completely.

Although these methods have alleviated the carrier phase delay problem to some extent, most fail to fundamentally solve the problem at the signal acquisition level. Moreover, under low signal-to-noise ratio or complex environmental interference conditions, the accuracy of delay estimation decreases, thereby undermining the stability of the entire demodulation process.

This study proposes a new method based on synchronous sampling with phase pre-calibration, which eliminates phase delay in signals at the source by adjusting the sampling point offset of the acquired interference signal. Specifically, the delay in the interference signal is first estimated algorithmically, then the starting point of the acquired data is adjusted according to the correspondence between sampling rate and carrier period, so that the adjusted signal contains negligible or minimal phase delay. This ensures maximum amplitude of the first and second harmonic cosine components in the mixed signal, fundamentally avoiding the denominator approaching zero problem. Compared with traditional compensation coefficient methods, this approach can avoid denominator singularity and reduce risks of nonlinear distortion and high-frequency oscillation. Unlike carrier phase correction-based methods, this technique performs phase pre-compensation at the data acquisition stage, completing correction of the interference signal at the acquisition level, thereby suppressing phase delay to near zero. It maintains compatibility with existing PGC demodulation architectures, requiring only the addition of a data preprocessing module without affecting other parameter settings in the demodulation process.

This method not only improves the overall performance of fiber optic interferometric measurement systems but also provides a new approach for optimizing phase delay compensation in complex application scenarios in the future.

## 2. Measurement Principles

### 2.1. The Carrier Phase Delay Problem in the PGC Algorithm

**Figure 1:**
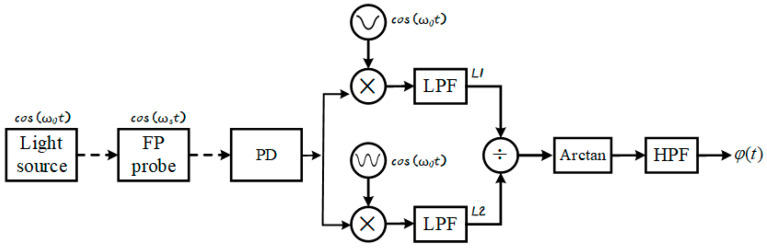
Schematic diagram of conventional PGC-Atan algorithm. The dashed arrow indicates optical signals, and the solid arrow indicates digital electrical signals.

The Phase Generated Carrier (PGC) demodulation technique achieves precise phase signal extraction through high-frequency carrier modulation. Its fundamental principle is illustrated in Figure 1.

Under drive current at frequency *ω*_0_, the laser generates optical frequency modulation, while the Fabry–Perot (FP) cavity length is modulated by the measurement signal at frequency *ω*_s_. The optical signal produced by low-finesse two-beam interference, after photoelectric conversion, can be expressed as

(1) $$V\left(t\right)=A+B\cos\left[C\cos\omega_{0}t+\varphi (t)\right],$$

where A represents the DC component of the interference signal, B denotes the signal amplitude related to incident light intensity and reflectivity of FP cavity’s two reflective surfaces. *C* = 2π*L*_0_Δ*ν*/*c* is the carrier phase modulation depth associated with static cavity length *L*_0_ and maximum optical frequency variation Δ*ν*. *ω*_0_ is the carrier frequency. *φ*(*t*) = *D*cos *ω*_s_*t* + *φ*_0_ contains the measurement signal phase and initial phase difference, where D represents the amplitude of measurement signal proportional to the optical center frequency and FP cavity length variation. *φ*_0_ is the static phase difference which, under ideal conditions, is solely determined by FP static cavity length and optical center frequency, but in practical systems may also be affected by environmental and other factors.

Applying Jacobi-Anger expansion to Equation (1), the frequency-modulated signal can be expanded into a series of Bessel functions and harmonic components as in Equation (2).

(2) $$\begin{array}{c} V\left(t\right)=A+B\left[\cos\left(C\cos\omega_{0}t\right)\cos\varphi \left(t\right)-\sin\left(C\cos\omega_{0}t\right)\sin\varphi \left(t\right)\right]\\ =A+B\left\{\begin{array}{l} J_{0}\left(C\right)\cos\varphi \left(t\right)\\ +2\sum_{k=1}^{\infty }{\left(-1\right)}^{k}J_{2k}\left(C\right)\cos2k\omega_{0}t\cos\varphi \left(t\right)\\ +2\sum_{k=1}^{\infty }{\left(-1\right)}^{k}J_{2k-1}\left(C\right)\cos\left(2k-1\right)\omega_{0}t\sin\varphi \left(t\right) \end{array}\right\} \end{array}$$

This expansion reveals a discrete harmonic structure in the signal spectrum centered at *nω*_0_, with each harmonic amplitude determined by Bessel function J_n_(*C*). The measurement phase information *φ*(*t*) is embedded in sideband structures of various harmonics, and the core of demodulation algorithm involves extracting specific harmonic sideband components (such as first and second order) through operations like mixing and filtering, thereby reconstructing *φ*(*t*).

By mixing with first and second harmonics of the carrier followed by low-pass filtering, the following two signals can be constructed:

(3) $$L_{1}=-BJ_{1}\left(C\right)\sin\left[\varphi \left(t\right)\right],$$

(4) $$L_{2}=-BJ_{2}\left(C\right)\cos\left[\varphi \left(t\right)\right].$$

Orthogonalization can be achieved either by selecting *C* such that J_1_(*C*) = J_2_(*C*), or by constructing a reciprocal multiplication coefficient J_2_(*C*)/J_1_(*C*). Phase difference can then be obtained by division and arctangent operation:

(5) $$\varphi \left(t\right)=\arctan\left[\frac{J_{2}\left(C\right)L_{1}\left(t\right)}{J_{1}\left(C\right)L_{2}\left(t\right)}\right].$$

In conventional PGC demodulation algorithms, the first and second harmonic signals are constructed as zero-phase reference signals, i.e., cos (*ω*_0_*t*) and cos (2*ω*_0_*t*). However, factors such as optical fiber transmission, environmental temperature drift, circuit group delay, and non-synchronization between reference carrier generation clock and signal sampling can all cause phase mismatch between reference carrier signals (first and second harmonics) and modulated carriers in actual interference signals. These are not ideal zero-phase differences, meaning the actual acquired interference signals contain an initial phase *θ*, and Equation (4) can be modified as

(6) $$V\left(t\right)=A+B\cos\left[C\cos\left(\omega_{0}t+\theta \right)+\varphi (t)\right].$$

After mixing with first and second harmonics and low-pass filtering, we obtain:

(7) $$L_{1}\left(t\right)=-BJ_{1}\left(C\right)\cos\theta \sin\left[\varphi \left(t\right)\right],$$

(8) $$L_{2}\left(t\right)=-BJ_{2}\left(C\right)\cos2\theta \cos\left[\varphi \left(t\right)\right].$$

The phase signal is still obtained through arctangent operation:

(9) $$\begin{array}{cc} \varphi \left(t\right) & =\arctan\left(\frac{J_{2}\left(C\right)L_{1}\left(t\right)}{J_{1}\left(C\right)L_{2}\left(t\right)}\right)\\ & =\arctan\left(\frac{\cos\theta \sin\varphi \left(t\right)}{\cos2\theta \cos\varphi \left(t\right)}\right)\\ & =\arctan\left(K\left(\theta \right)\tan\varphi \left(t\right)\right) \end{array}.$$

The compensation coefficient *K*(*θ*) is defined as the ratio of orthogonal components (*L*_1_(*t*) and *L*_2_(*t*)) after eliminating the influence of carrier modulation depth *C*, scaled by the carrier phase delay compensation term. From Equation (9), it can be observed that in arctangent demodulation algorithm, *θ* destroys the orthogonality between *L*_1_(*t*) and *L*_2_(*t*), introducing periodic harmonic errors into the extracted phase *φ*(*t*). These errors manifest as baseline drift or ripple noise in the demodulated signal.

A common method to estimate *θ* is to construct first harmonic sine product signal:

(10) $$\begin{array}{cc} VS_{1}\left(t\right) & =V\left(t\right)\sin\omega_{0}t\\ & =A\sin\omega_{0}t+B\cos\left[C\cos\left(\omega_{0}t+\theta \right)+\varphi (t)\right]\sin\omega_{0}t. \end{array}$$

After low-pass filtering, the first harmonic sine mixed signal is obtained:

(11) $$LS_{1}\left(t\right)=BJ_{1}\left(C\right)\sin\theta \sin\left[\varphi \left(t\right)\right].$$

Dividing Equation (7) by Equation (11) yields:

(12) $$\frac{\sin\theta }{\cos\theta }=-\frac{LS_{1}\left(t\right)}{L_{1}\left(t\right)}.$$

Current research typically uses Equation (12) to determine *θ* value, which is then substituted into Equations (7) and (8) to further solve for *φ*(*t*).

Studies have found that although this delay compensation method can partially suppress *θ*’s influence, it still fails in extreme phase delay scenarios. When *θ*→90°, cos *θ*→0, causing the amplitude of *L*_1_ to approach zero and the signal-to-noise ratio to deteriorate drastically. When *θ*→45°, cos 2*θ*→0, compensation coefficient *K* diverges, noise is infinitely amplified, leading to demodulation failure.

### 2.2. Signal Discretization Model and Phase Offset Mechanism

In interferometric measurement systems, the ideal reference carrier can be expressed as

(13) $$S_{ref}\left(t\right)=\cos\left(\omega_{0}t\right).$$

The modulated carrier in actual interference signals incorporates a phase offset *θ*, resulting from hardware delays and sampling clock mismatches:

(14) $$S_{\mathrm{mod}}\left(t\right)=\cos\left[\omega_{0}\left(t+t_{total}\right)\right]=\cos\left(\omega_{0}t+\theta \right),$$

where *t_total_* = *t_PD_* + *t_circuit_* + *t_ADC_* includes the photodetector response time *t_PD_*, analog channel group delay *t_circuit_*, and ADC conversion delay *t_ADC_*.

After photoelectric conversion, the interference signal is discretized by an ADC at sampling rate *f*_s_ and carrier frequency *f*_0_, yielding the observed sequence:

(15) $$V\left[n\right]=A+B\cos\left[C\cos\left(2\pi \frac{f_{0}}{f_{s}}n+\theta \right)+\varphi \left[n\right]\right],n=0,1,\cdots ,N-1,$$

where *θ* is the static phase offset of this observation sequence, and *N* is the number of sampling points per measurement.

**Figure 2:**
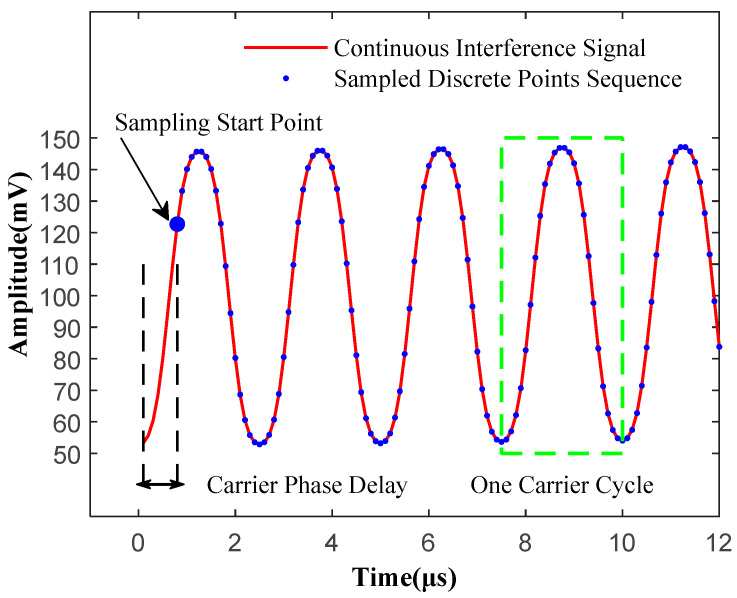
Interference Signal Observation Sequence.

Figure 2 illustrates the relationship between the continuous interference signal and its discretized observation sequence. In this example, the analog carrier signal has a frequency of f_0_ = 400 kHz, while the sampling rate is *f*_s_ = 10 MHz. The green rectangular frame highlights one complete carrier cycle, within which approximately *f*_s_/*f*_0_ = 25 sampling points are visible, as calculated by Equation (16). Note that *N_cycle_* in Equation (16) may be non-integer, and sampling points may span multiple cycles.

(16) $$N_{\mathrm{cy}cle}=\frac{f_{s}}{f_{0}}.$$

Owing to the arbitrary starting time of sampling, the first acquired sample (marked with a solid blue dot in Figure 2 exhibits a fixed phase delay *θ* relative to the signal’s zero-time reference. This delay, visually emphasized by the two dashed vertical lines between the ideal signal origin and the first acquired sample, quantitatively represents the temporal offset introduced during acquisition.

The nominal phase increment per sampling point is:

(17) $$\Delta \phi_{per\text{-}sample}=\frac{f_{0}}{f_{s}}\cdot 2\pi .$$

For an estimated phase delay *θ*, the equivalent number of sampling points to shift is:

(18) $$\Delta n=\left\lfloor \frac{\theta }{\Delta \phi_{per\text{-}sample}}\right\rfloor =\left\lfloor \frac{\theta }{2\pi }\cdot \frac{f_{s}}{f_{0}}\right\rfloor .$$

In Equation (18), the shift point count Δ*n* is determined by rounding to the nearest integer. For instance, with *f*_s_ = 10 MHz and *f*_0_ = 400 kHz, if *θ* = 1.25 rad, then Δ*n* = 5.

A new signal sequence is then constructed starting from the Δ*n*-th sample:

(19) $$V_{new}\left[m\right]=V\left[m+\Delta n\right],m=0,1,\cdots ,N-\Delta n-1.$$

This operation is equivalent to time-shifting the signal to align the actual carrier phase with the reference carrier. The remaining carrier phase delay in the new sequence is defined as the residual delay Δ*θ*:

(20) $$\Delta \theta =\theta -\Delta n\Delta \phi_{per\text{-}sample}.$$

In the example above, the original delay was 1.25 rad. After a 5-point shift, the residual delay Δ*θ* is reduced to 0.0066 rad. This negligible value (Δ*θ* < 0.01 rad) indicates that the carrier phase is now effectively aligned with the ideal zero-phase state, achieving near-complete synchronization.

### 2.3. Sampling Rate Requirements for Complete Alignment

Phase delay compensation is achieved by shifting sampling points. Given a sampling rate of *f*_s_ = 10 MHz and a carrier frequency of *f*_0_ = 400 kHz, Equation (17) yields a per-sample phase interval of Δ*ϕ*_per-sample_ = 0.2513 rad. This implies that shifting by one sample corresponds to a phase adjustment of approximately 0.2513 radians. The resolution of the compensation is inversely proportional to this value—a smaller Δ*ϕ*_per-sample_ allows finer phase alignment. The maximum residual phase error after shifting is theoretically bounded to half of the phase interval per sample:

(21) $$\Delta \theta_{\max}=\frac{\Delta \phi_{per\text{-}sample}}{2}=\frac{f_{0}}{f_{s}}\cdot \pi .$$

The estimated phase obtained through the ratio method in Equation (5) is:

(22) $$\hat{\varphi }\left(t\right)=\arctan\left(\frac{L_{1}}{L_{2}}\right)=\arctan\left(\frac{\sin\varphi \left(t\right)}{\cos\varphi \left(t\right)}\cdot \frac{\cos\Delta \theta }{\cos2\Delta \theta }\right).$$

Under near-aligned conditions where *θ* = Δ*θ* ≈ 0, a first-order Taylor expansion yields:

(23) $$\cos\Delta \theta \approx 1-\frac{\Delta \theta^{2}}{2},\cos2\Delta \theta \approx 1-2\Delta \theta^{2}.$$

Thus, the ratio simplifies to

(24) $$\frac{L_{1}}{L_{2}}=\frac{\cos\Delta \theta }{\cos2\Delta \theta }\cdot \tan\varphi \left(t\right)\approx \left(1+\frac{3\Delta \theta^{2}}{2}\right)\cdot \tan\varphi \left(t\right).$$

Therefore, the phase estimation error is

(25) $$\delta \varphi \left(t\right)=\hat{\varphi }\left(t\right)-\varphi \left(t\right)\approx \arctan\left[\left(1+\frac{3\Delta \theta^{2}}{2}\right)\tan\varphi \left(t\right)\right]-\varphi \left(t\right).$$

Let

(26) $$\varepsilon =\frac{3\Delta \theta^{2}}{2}.$$

Further simplification via Taylor expansion:

(27) $$\arctan\left[\left(1+\varepsilon \right)\tan\varphi \left(t\right)\right]\approx \varphi \left(t\right)+\varepsilon \cdot \frac{\sin\varphi \left(t\right)\cos\varphi \left(t\right)}{1+\varepsilon \sin^{2}\varphi \left(t\right)}.$$

For ε ≪ 1, the phase estimation error approximates

(28) $$\delta \varphi \left(t\right)\approx \varepsilon \cdot \sin\varphi \left(t\right)\cos\varphi \left(t\right)=\frac{3\Delta \theta^{2}}{2}\cdot \sin\varphi \left(t\right)\cos\varphi \left(t\right).$$

The error amplitude depends on sin φ(t)cos *φ*(*t*), with maximum value 1/2 (when φ(t) = ±π/4). Hence, the maximum phase error is

(29) $${\left|\delta \varphi \left(t\right)\right|}_{\max}\approx \frac{3\Delta \theta^{2}}{4}.$$

If the required phase error does not exceed *δ*_max_, then

(30) $$\Delta \theta \leq \sqrt{\frac{4\delta_{\max}}{3}}.$$

For a displacement resolution of 0.1 nm, the corresponding radian precision is approximately 0.0008 rad. Substituting into Equation (31) gives Δ*θ* ≤ 0.033 rad. This constraint requires the phase increment per sample to be below approximately 0.066 rad, implying at least 96 samples per carrier cycle. With f_0_ = 400 kHz, the sampling rate needs to reach 38.4 MHz.

The computational load of the demodulation process includes:

(1) Mixing: Each sampling point multiplied with carrier (sine/cosine)—2 multiplications
(2) Low-pass filtering: Assuming *M*-th order FIR filter—2 *M* multiply-accumulate operations per point (counted as 1 operation each)
(3) Arctangent operation: Using approximation algorithms or lookup tables—about 10 operations per point

The computational load can be expressed in Million Operations Per Second (MOPS):

(31) $$MOPS=\frac{f_{s}\times \left(2+2M+10\right)}{10^{6}}$$

For *M* = 500 order filter, reducing f_s_ from 10 MHz to 5 MHz decreases MOPS from 1120 to 560 (50% reduction), while maintaining errors within allowable range and achieving linear computation reduction.

At the reduced sampling rate of 3.84 MHz (one order of magnitude lower), the maximum residual phase delay Δ*θ*_max_, as derived from Equation (21), is 0.33 rad. Under this condition, the amplitude coefficients cos *θ* and cos 2*θ* are approximately 0.95 and 0.79, respectively, resulting in a maximum signal amplitude attenuation of less than 21%. This performance is considered acceptable in practical applications, as it effectively avoids demodulation failures associated with critical phase values. Should further improvement in signal-to-noise ratio (SNR) be required, the sampling rate can be increased to further reduce the residual phase error Δ*θ*.

Based on this analysis, we designed a delay phase pre-alignment compensation algorithm, which can effectively avoid the near-zero compensation coefficient problem caused by θ singular points when using relatively low sampling rates.

### 2.4. Pre-Alignment and Coefficient Compensation Co-Optimization

**Figure 3:**
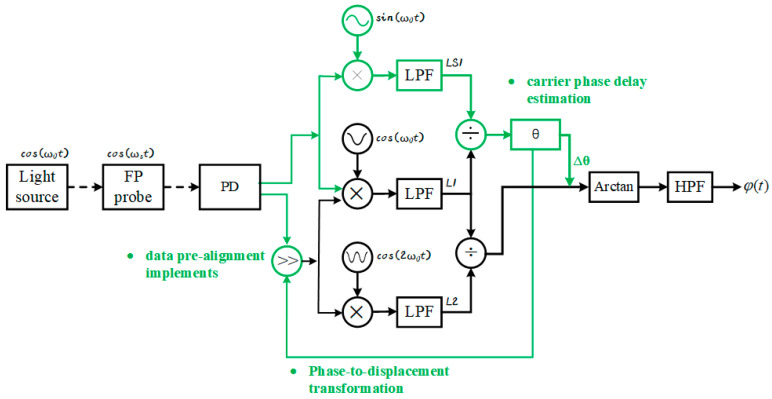
Flowchart of the delay phase pre-alignment compensation algorithm. The dashed arrow indicates optical signals. The modifications introduced by the algorithm proposed in this paper, compared to the traditional PGC demodulation algorithm, are highlighted in green.

The structure of the delay phase pre-alignment compensation algorithm is depicted in Figure 3.

The green section in Figure 3 delineates the innovative pre-alignment compensation module proposed in this study. This module comprises three pivotal computational stages executed prior to conventional PGC demodulation. Firstly, the carrier phase delay *θ* is estimated using Equation (12), which calculates *θ* through orthogonal phase-locked demodulation of the first-harmonic reference signals. Subsequently, Equation (18) converts the continuous phase delay into discrete sampling-point shifts by computing Δ*n*. This phase-to-displacement transformation quantizes the analog delay into integer-sample operations. Finally, data pre-alignment implements the phase compensation at the acquisition level by shifting the sampled sequence by Δ*n* points. This operation bounds the residual error within Δ*θ*.

By preprocessing signals ahead of conventional PGC demodulation, this module fundamentally eliminates critical failure points (e.g., *θ* = π/4 where cos 2*θ*→0). Residual phase errors are algorithmically constrained away from singularity regions. The architectural innovation shifts computation-intensive real-time compensation to a single-initialization operation, reducing processing overhead while maintaining compatibility with existing PGC frameworks.

## 3. Experimental Analysis

**Figure 4:**
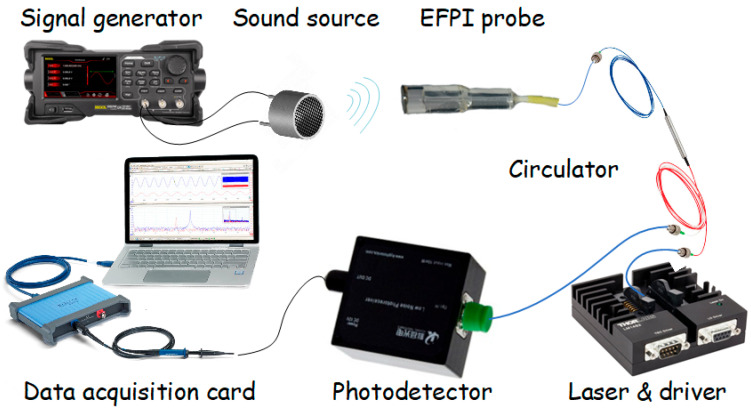
Schematic of the EFPI signal demodulation system.

To validate the feasibility of the complete solution, long-term demodulation stability tests were performed on EFPI ultrasonic sensors. A schematic of the experimental setup is shown in Figure 4. The light source was a DFB laser (BFLD-155010-SM-FA, Box, Shenzhen, China) with a center wavelength of 1550.23 nm and a modulation sensitivity of 0.01 nm/mA. Optical frequency modulation was applied using a custom laser driver circuit operating at 500 kHz with a modulation current of 100 mA ± 60 mA. The laser output was injected into port 1 of a fiber circulator, with port 2 connected to the EFPI probe (M100, Shanghai University, Shanghai, China). The interference light reflected from the Fabry–Perot cavity carried phase difference information induced by external acoustic waves on the sensitive diaphragm, which was output through port 3 to a photodetector (PIN photodetector module, Keyang, Beijing, China) for conversion into electrical signals. These signals were then sampled at 10 MS/s by a data acquisition card (4225A, Pico Technology, UK) and transferred to a computer for demodulation. A piezoelectric ceramic and metal composite vibrator (HC40C16TR-1, HC Sensors, Shenzhen, China) was used as the acoustic source, driven by a 40 kHz sinusoidal signal from a function generator. The drive voltage was held constant throughout the measurements.

**Figure 5:**
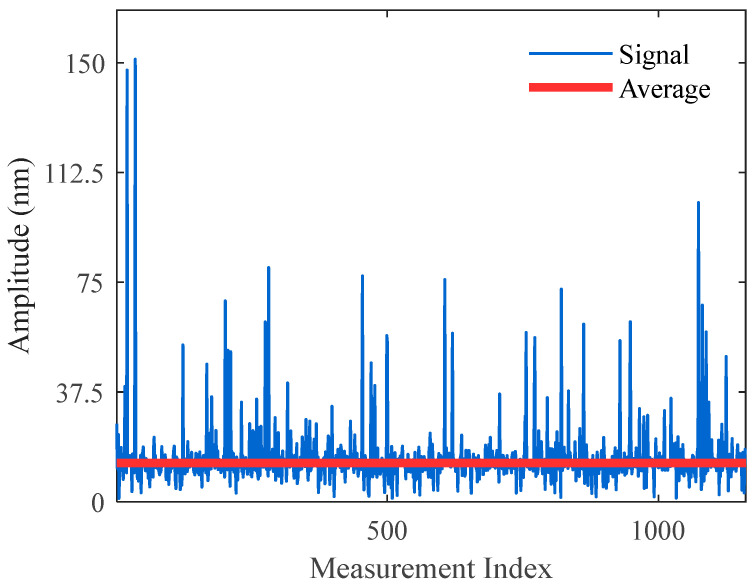
Demodulation results using the traditional phase compensation algorithm.

**Figure 6:**
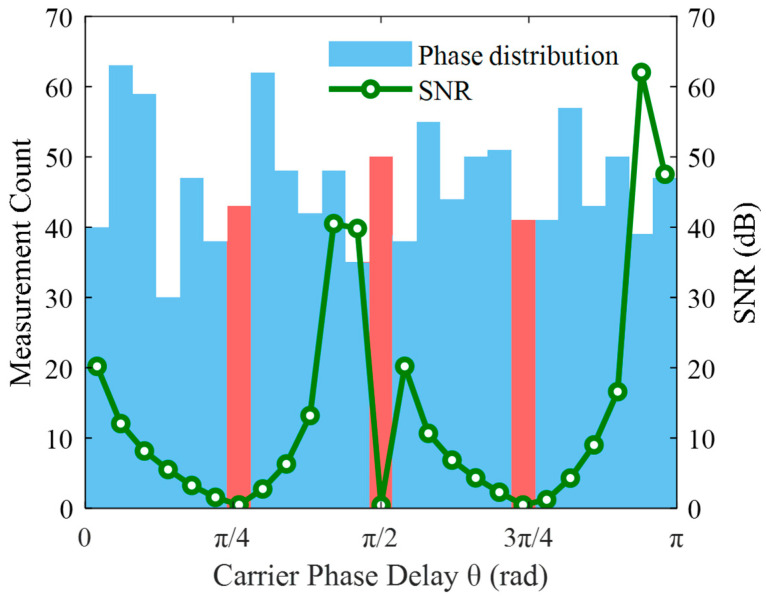
Phase delay distribution in the traditional phase compensation algorithm. The red areas indicate the phase delay regions where cos *θ* or cos 2*θ* becomes zero.

Throughout the measurement period, the acoustic pressure signal remained unchanged. A total of 1100 measurements were taken at 60-s intervals. First, the traditional phase compensation algorithm was used for demodulation, where phase delay was calculated using Equation (12), the coefficient *K* was constructed, and then phase *φ*(*t*) was computed. The demodulation results are shown in Figure 5. Although this method yielded relatively accurate average values over long-term measurements, significant random deviations occurred in some demodulation outcomes. The distribution of phase delays across the 1100 measurements is illustrated in Figure 6. Due to phase ambiguity, phase delays were limited to the range of [0, π] and divided equally into 25 intervals. That is, phase delays between 0~π/25 fell into statistical interval ①, phase delays between π/25~2π/25 fell into interval ②, and so on. The phase delays were basically uniformly distributed. By evaluating the mean square error of demodulated results within each interval and computing the narrow-band signal-to-noise ratio (SNR), it was observed that the SNR approached zero near phase delays of π/4, π/2, and 3π/4, indicating demodulation failure.

**Figure 7:**
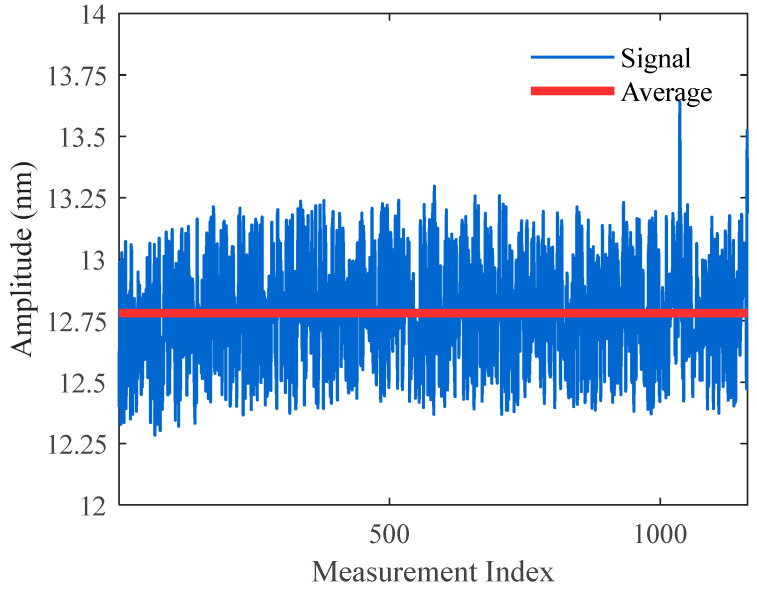
Demodulation results using the pre-alignment demodulation algorithm.

**Figure 8:**
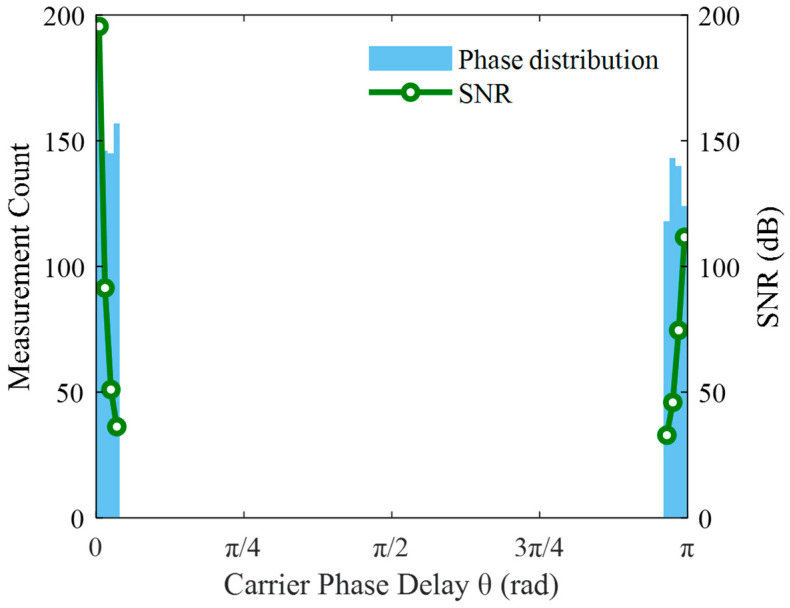
Phase delay distribution in the pre-alignment demodulation algorithm.

Under the same experimental conditions, the proposed shifted pre-alignment demodulation algorithm was implemented. The corresponding demodulation results and phase delay distributions are presented in Figure 7 and Figure 8, respectively.

Comparing Figure 6 and Figure 8 reveals that in the traditional demodulation algorithm, phase delays were approximately uniformly distributed between 0~2π. Demodulation failure occurred near π/4, π/2, and 3π/4, where the SNR approached zero. In contrast, in Figure 8 using the pre-alignment demodulation algorithm, phase delays were mainly concentrated at 0 or π. At these values, both cos *θ* and cos 2*θ* are close to 1, completely avoiding demodulation failure. The minimum SNR of demodulated signal amplitudes reached 35 dB, indicating a 35 dB improvement in worst-case SNR compared to the traditional method.

A direct comparison of the demodulation results in Figure 6 and Figure 8 clearly demonstrates the enhanced stability offered by the pre-alignment algorithm. The standard deviation of the demodulated vibration amplitude decreases from 10.1 nm to 0.2 nm, representing a 50.5-fold enhancement in measurement consistency. This reduction in fluctuation translates to more reliable analysis of physical variations, fewer false alarms, and significantly improved system robustness in practical applications.

## 4. Conclusions

This study overcomes the challenges of nonlinear distortion and signal-to-noise ratio (SNR) degradation in Fabry–Perot (FP) sensor phase-generated carrier (PGC) demodulation caused by carrier phase delay. We propose a phase delay compensation method based on sampling-point shift pre-alignment and demonstrate its key performance benefits through both theoretical modeling and experimental validation.

The proposed sampling-point shift pre-alignment algorithm dynamically adjusts the starting point of the sampling sequence to achieve discrete carrier phase alignment, reducing residual phase error to the theoretical limit of π*f*_0_/*f*_s_. Furthermore, we derive the nonlinear distortion factor ϵ = 3Δ*θ*^2^/2, demonstrating that a sampling rate *f*_s_ ≥ 3.84 MHz limits the maximum signal amplitude attenuation to below 21%, effectively eliminating *θ*-induced demodulation failures and meeting the demands of high-precision measurement.

Compared to traditional real-time compensation methods, this algorithm avoids computational singularities due to denominator zero-crossing and improves demodulation SNR by two orders of magnitude at *θ* = 45°. By implementing phase compensation through a preprocessing module, the method circumvents the real-time bottlenecks typical of iterative strategies. The proposed method is compatible with existing PGC demodulation architectures, requiring no modifications to filter parameters or carrier generation modules.

Experimental validation on an EFPI sensor under 40 kHz ultrasonic excitation showed that the minimum demodulation SNR improved from 0 dB (traditional method) to 35 dB. In 1100 long-term test datasets, the standard deviation of the demodulated vibration amplitude of the sensitive diaphragm decreased from 10.1 nm (traditional method) to 0.2 nm, with no demodulation failures observed at phase delay-sensitive points, achieving a 50.5-fold improvement in stability

Future work will focus on multi-channel delay synchronization calibration techniques and adaptive sampling rate control mechanisms to further expand its application potential in critical engineering fields such as partial discharge monitoring in power equipment, deep-sea exploration, and aero-engine health monitoring.

## Data Availability

Data are contained within the article.
